# Supplementary figures and images for: IFIT1 is rapidly evolving and exhibits disparate antiviral activities across 11 mammalian orders
Source: eLife. 2025 Oct 22;13:RP101929. doi: 10.7554/eLife.101929 (PMC12543323; doi:10.7554/eLife.101929)

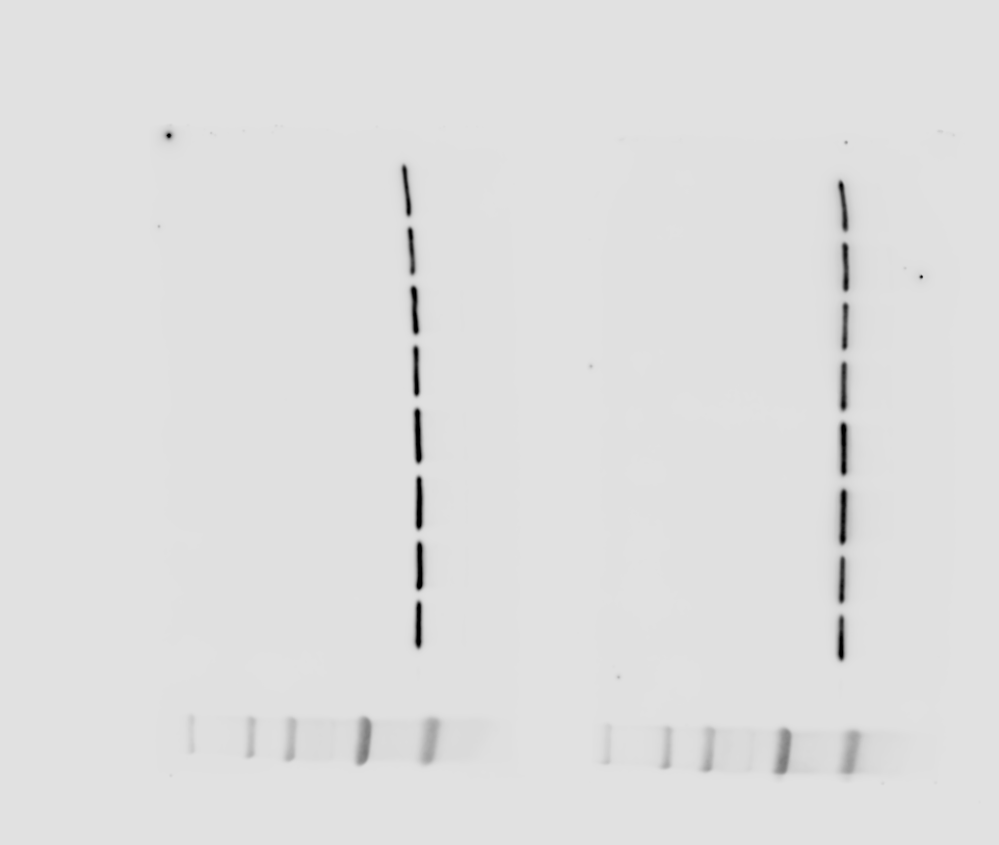

Supplement: Figure 2—source data 2. [file elife-101929-fig2-data2.zip › Figure2-SourceData-2/2024.04.12_ GAPDH - Mem 1 and 2.tif]

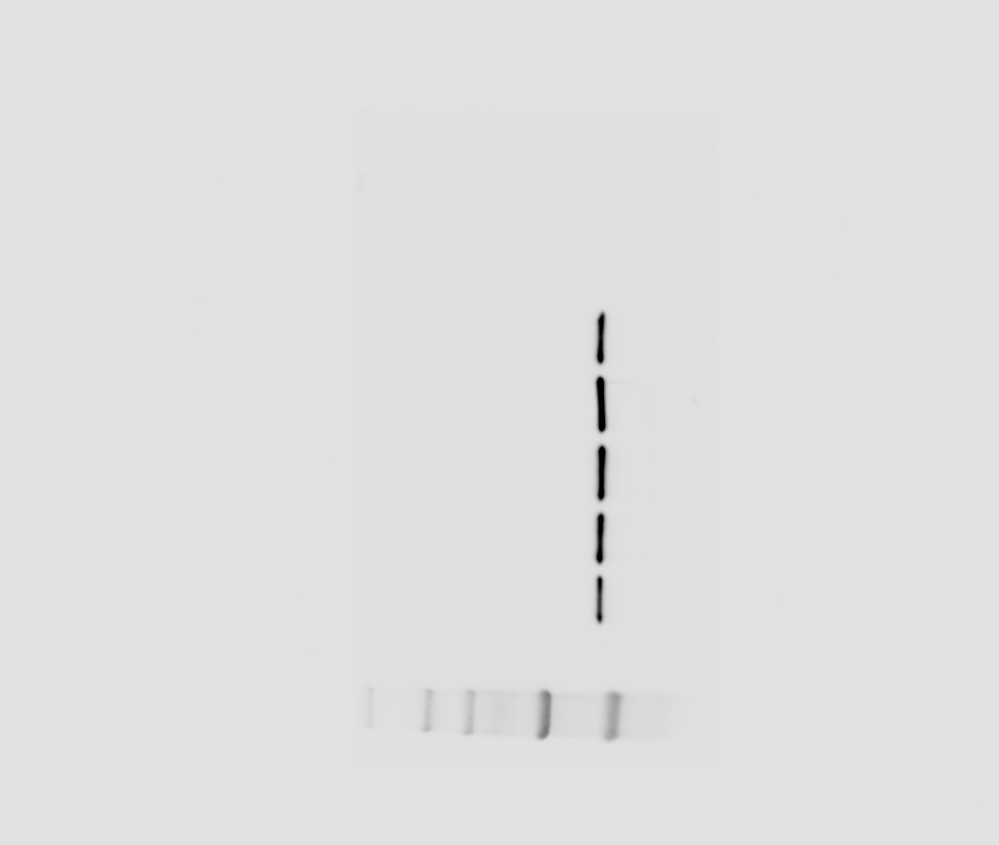

Supplement: Figure 2—source data 2. [file elife-101929-fig2-data2.zip › Figure2-SourceData-2/2024.04.12_ GAPDH - Mem 3.tif]

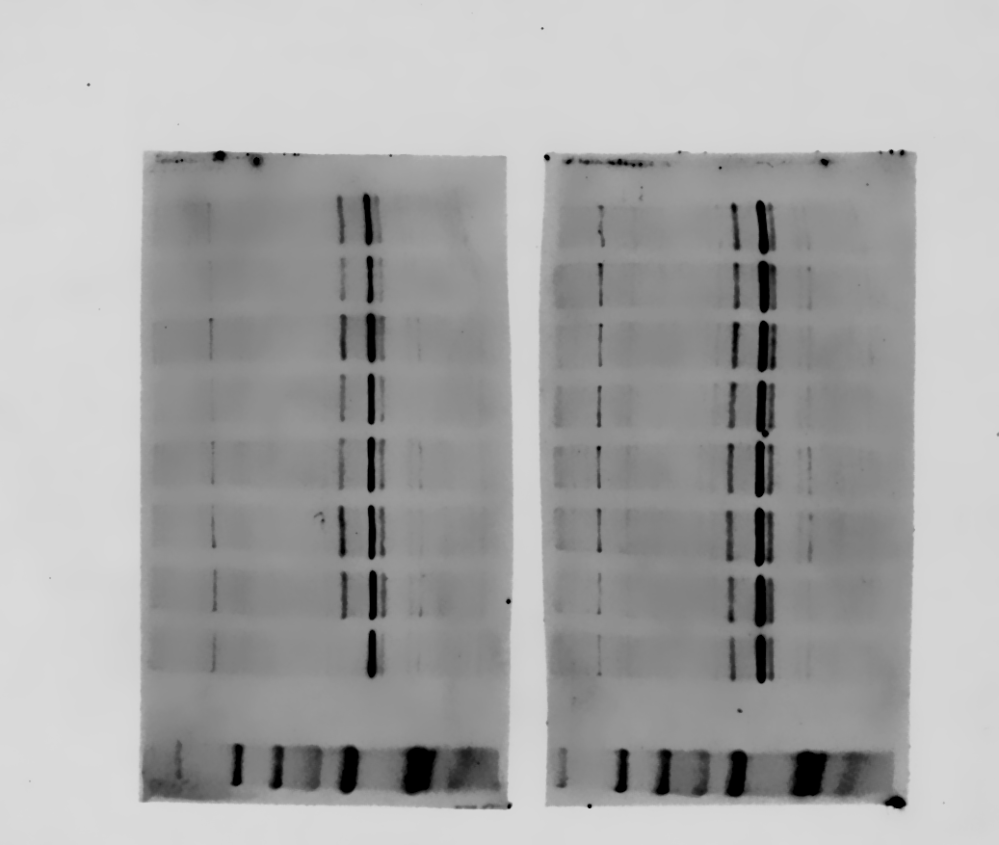

Supplement: Figure 2—source data 2. [file elife-101929-fig2-data2.zip › Figure2-SourceData-2/2024.04.12_ IFIT1 - Mem 1 and 2.tif]

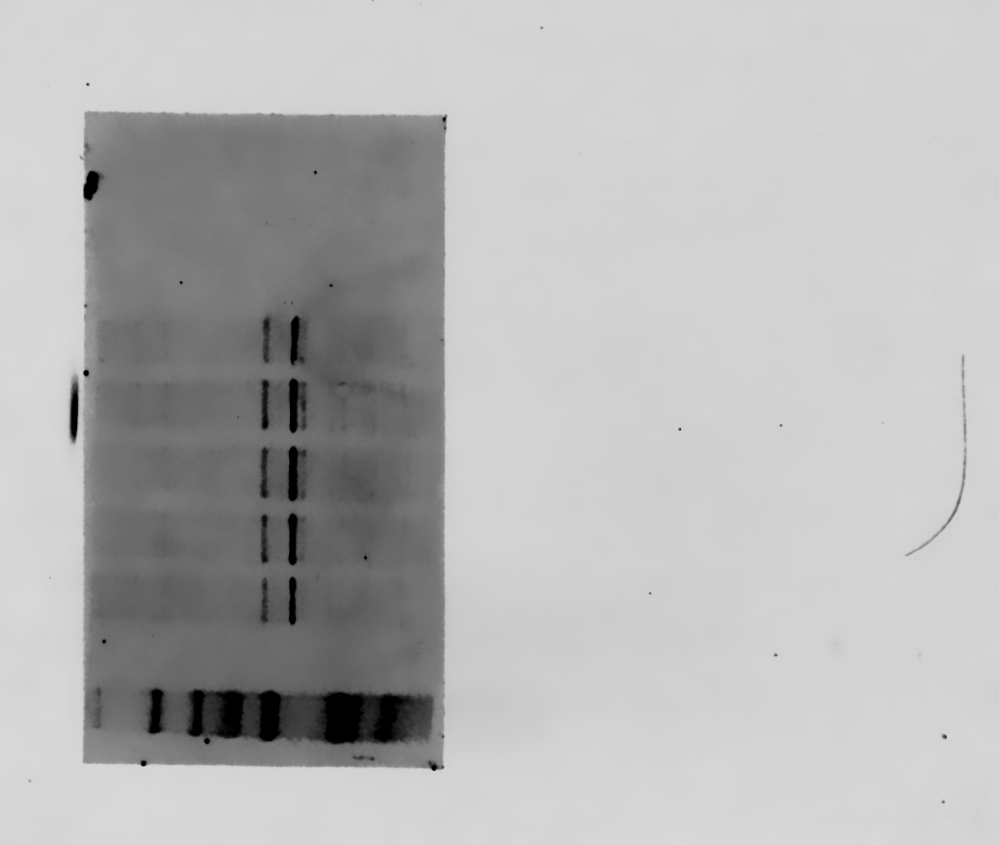

Supplement: Figure 2—source data 2. [file elife-101929-fig2-data2.zip › Figure2-SourceData-2/2024.04.12_ IFIT1 - Mem 3.tif]

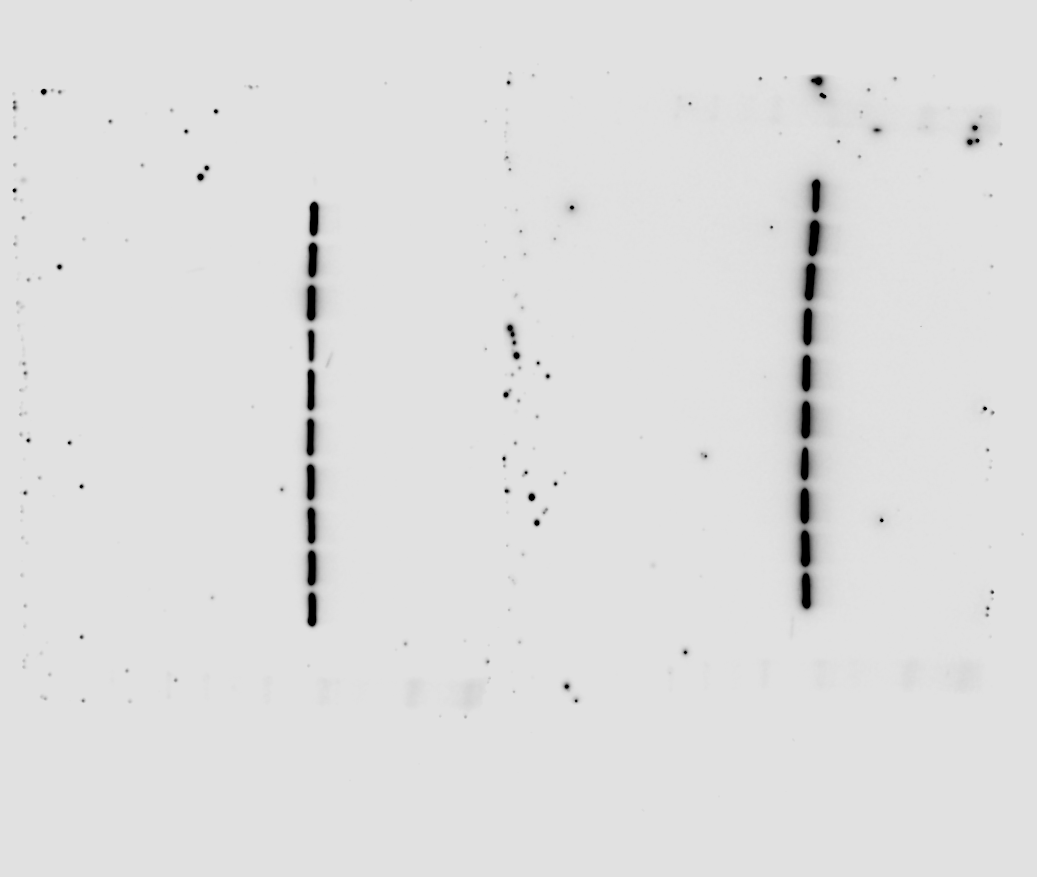

Supplement: Figure 3—figure supplement 1—source data 2. [file elife-101929-fig3-figsupp1-data2.zip › Figure3-FigureSupplement1-SourceData-2/23.09.13.IFIT1OrthScreen.antiGAPDH.B7toC5leftandC6toD3Right.tif]

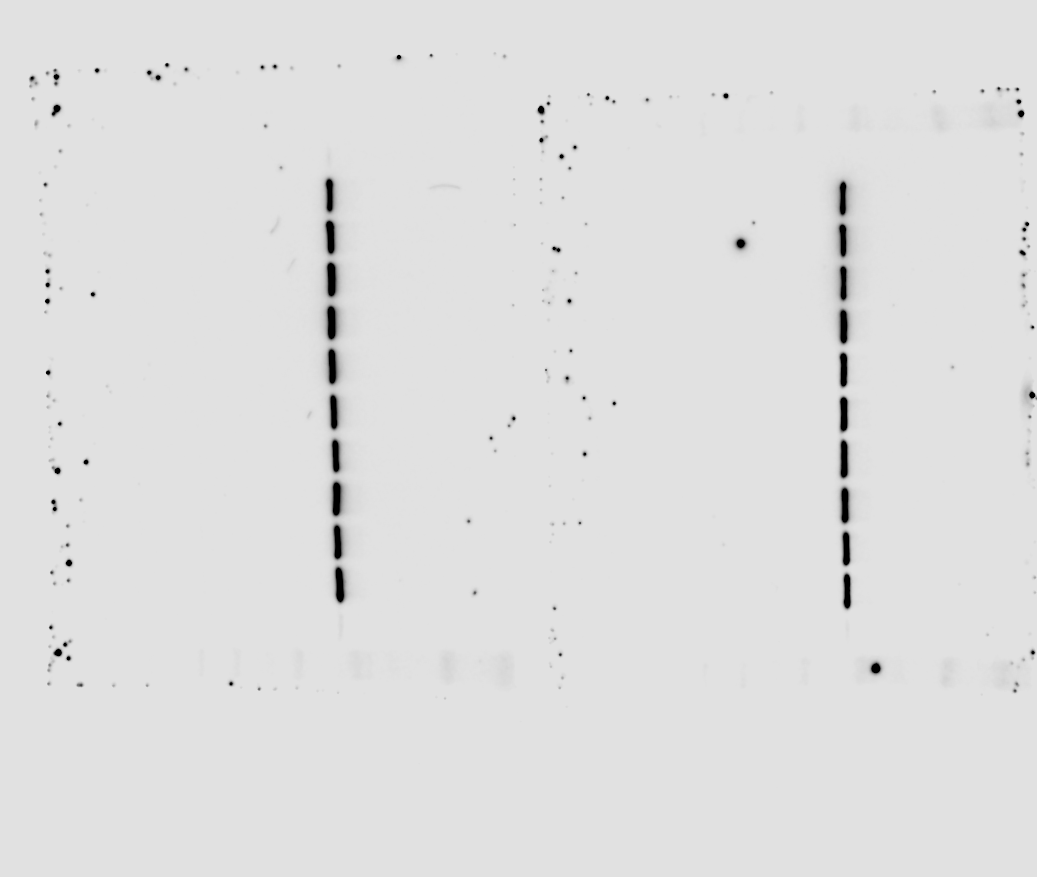

Supplement: Figure 3—figure supplement 1—source data 2. [file elife-101929-fig3-figsupp1-data2.zip › Figure3-FigureSupplement1-SourceData-2/23.09.13.IFIT1OrthScreen.antiGAPDH.EmptoA7LeftandA9toB6.tif]

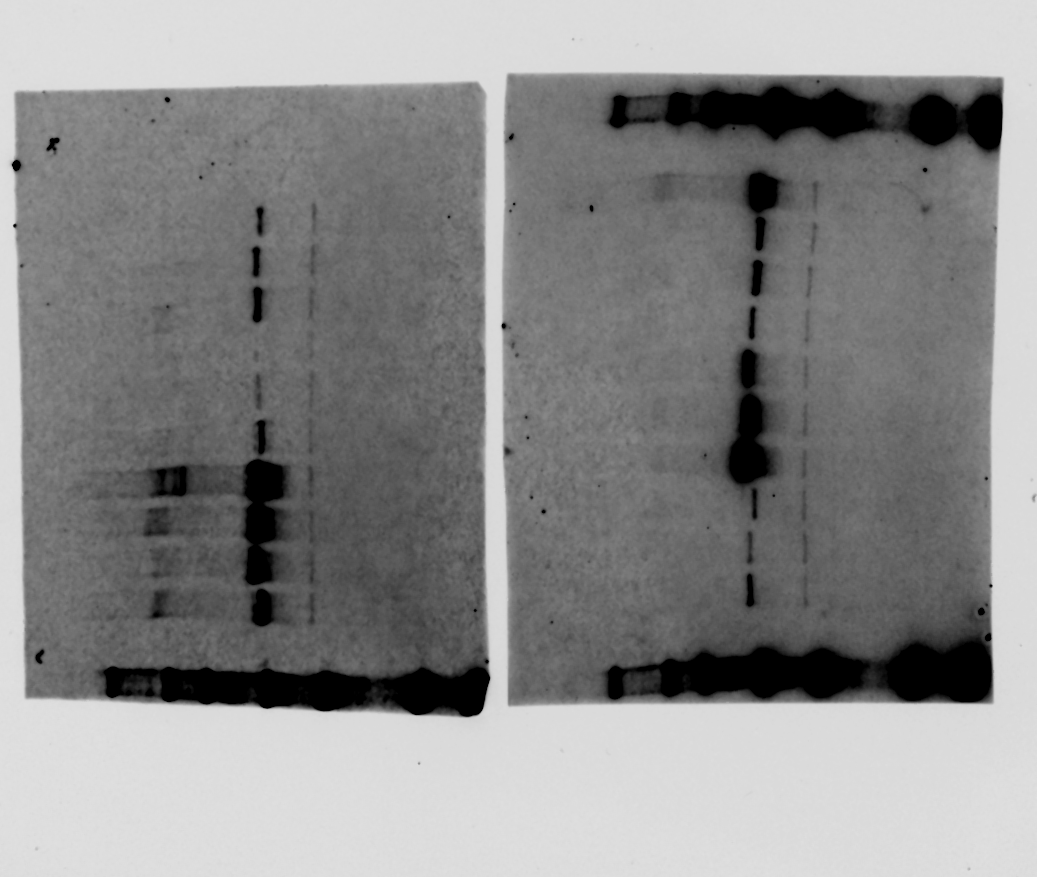

Supplement: Figure 3—figure supplement 1—source data 2. [file elife-101929-fig3-figsupp1-data2.zip › Figure3-FigureSupplement1-SourceData-2/23.09.13.IFIT1OrthScreen.antiHA.B7toC5leftandC6toD3Right.tif]

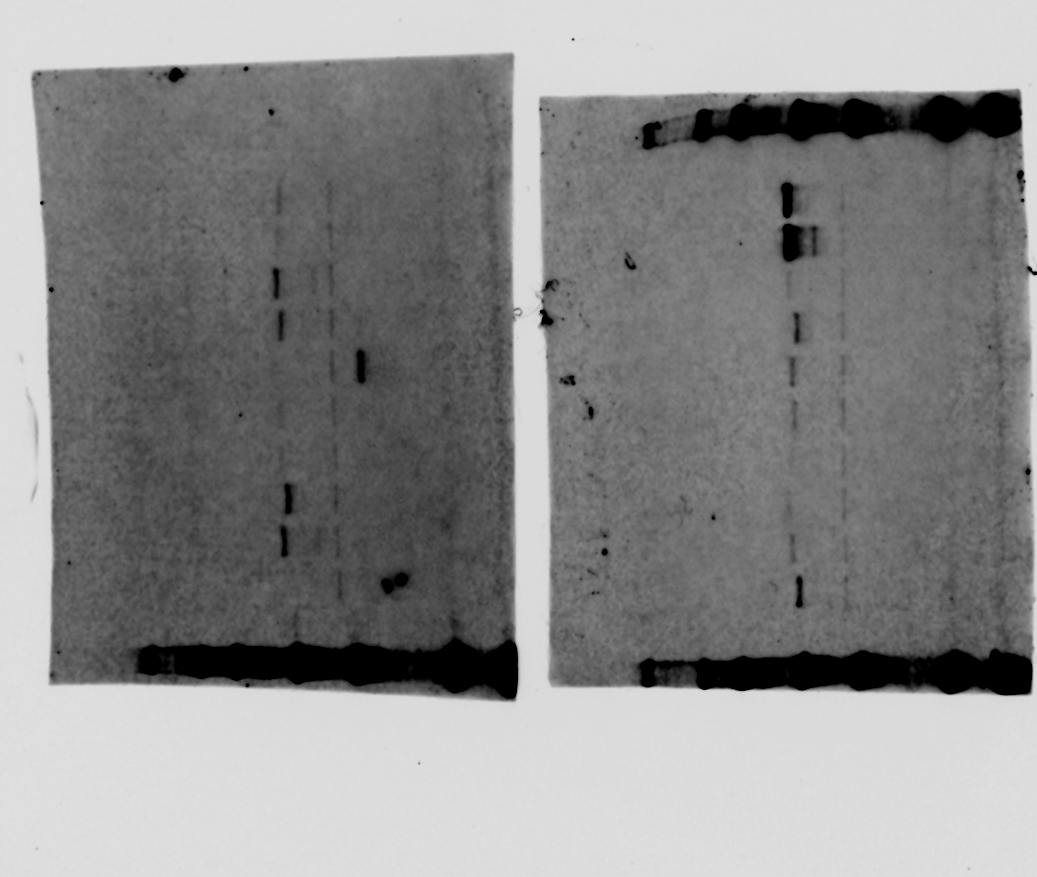

Supplement: Figure 3—figure supplement 1—source data 2. [file elife-101929-fig3-figsupp1-data2.zip › Figure3-FigureSupplement1-SourceData-2/23.09.13.IFIT1OrthScreen.antiHAEmptoA7LeftandA9toB6.tif]

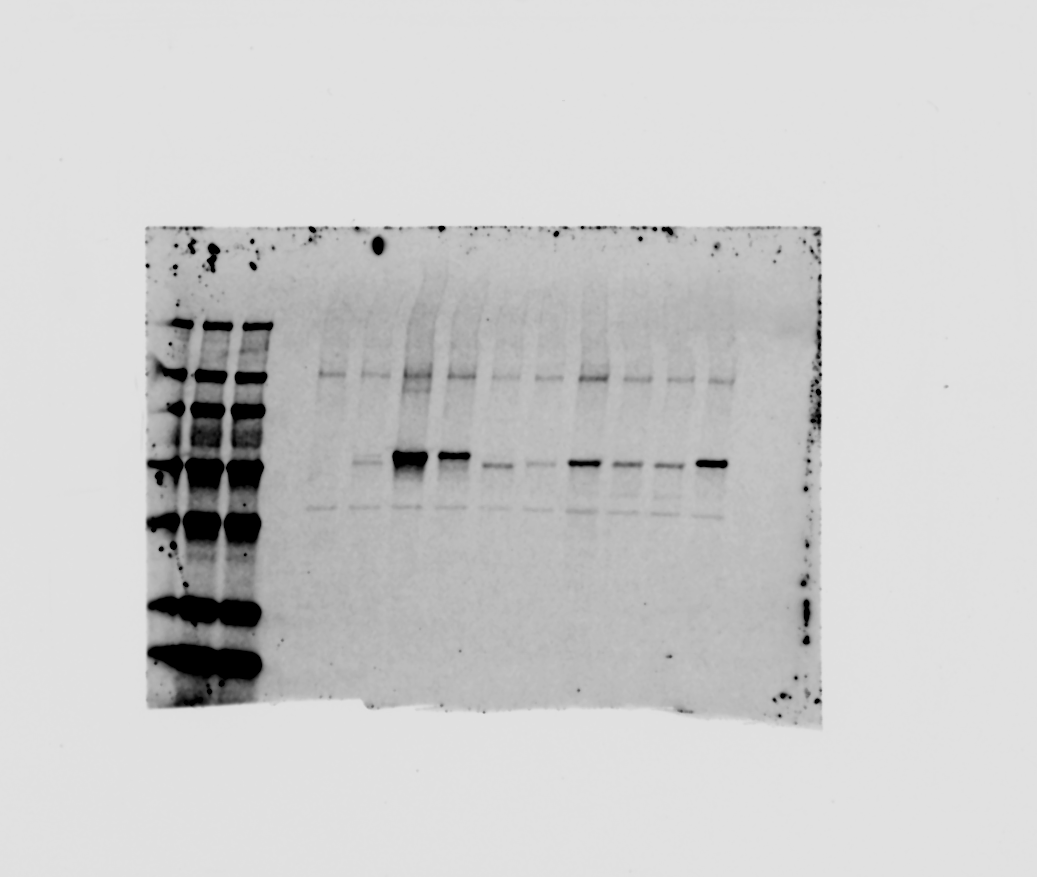

Supplement: Figure 4—source data 2. [file elife-101929-fig4-data2.zip › Figure4-SourceData-2/23.08.15_MsantiHA.tif]

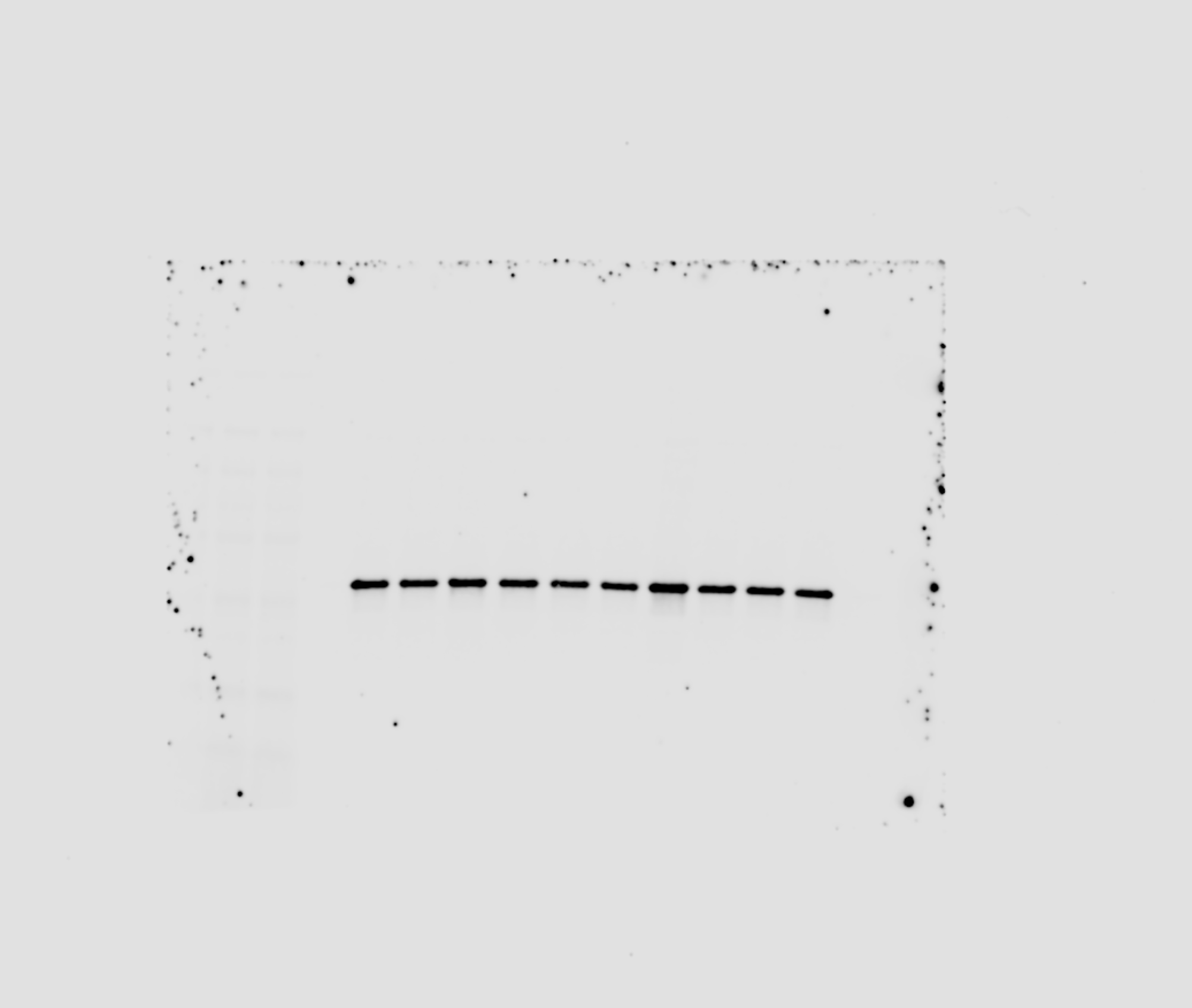

Supplement: Figure 4—source data 2. [file elife-101929-fig4-data2.zip › Figure4-SourceData-2/23.08.15_RbantiGAPDH.tif]

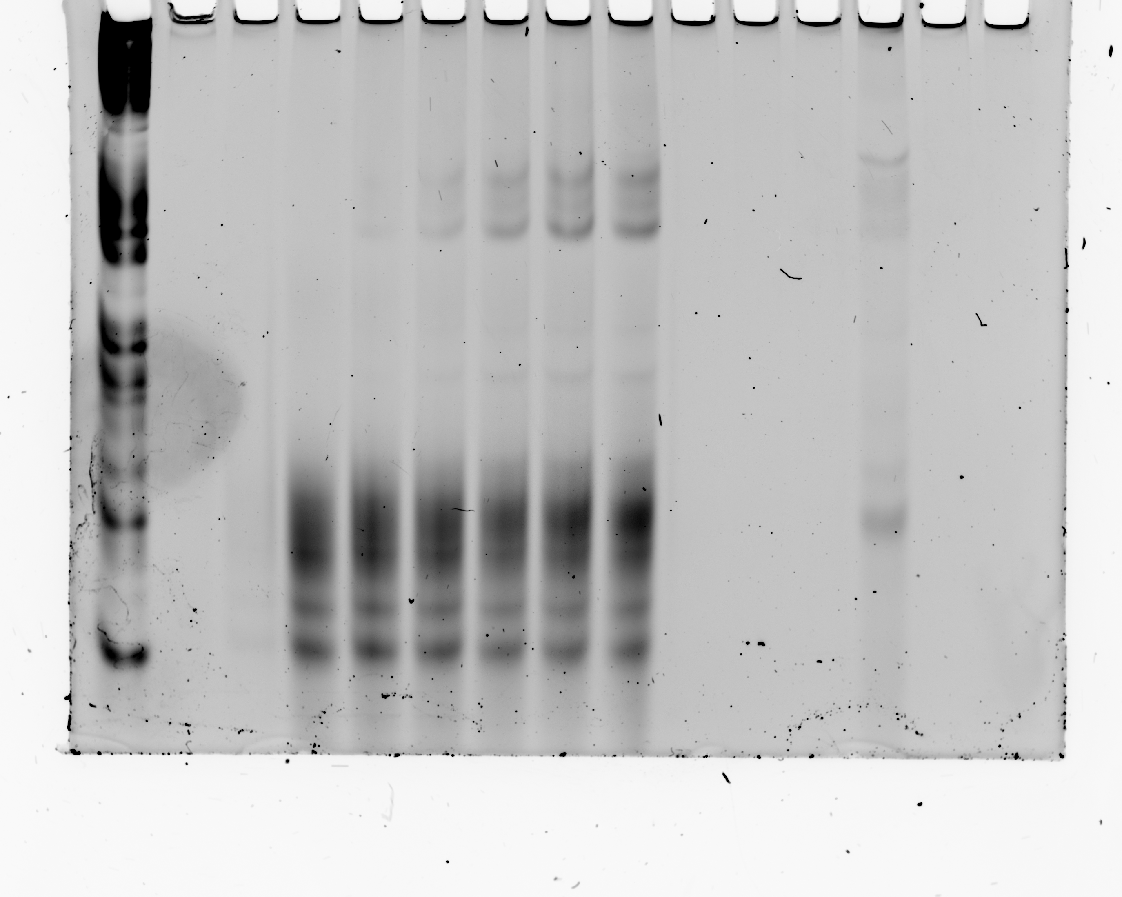

Supplement: Figure 5—source data 2. [file elife-101929-fig5-data2.zip › Figure5-SourceData-2/Schoggins Lab 2023-12-29 18h13m05s(SYBR® Gold).tif]

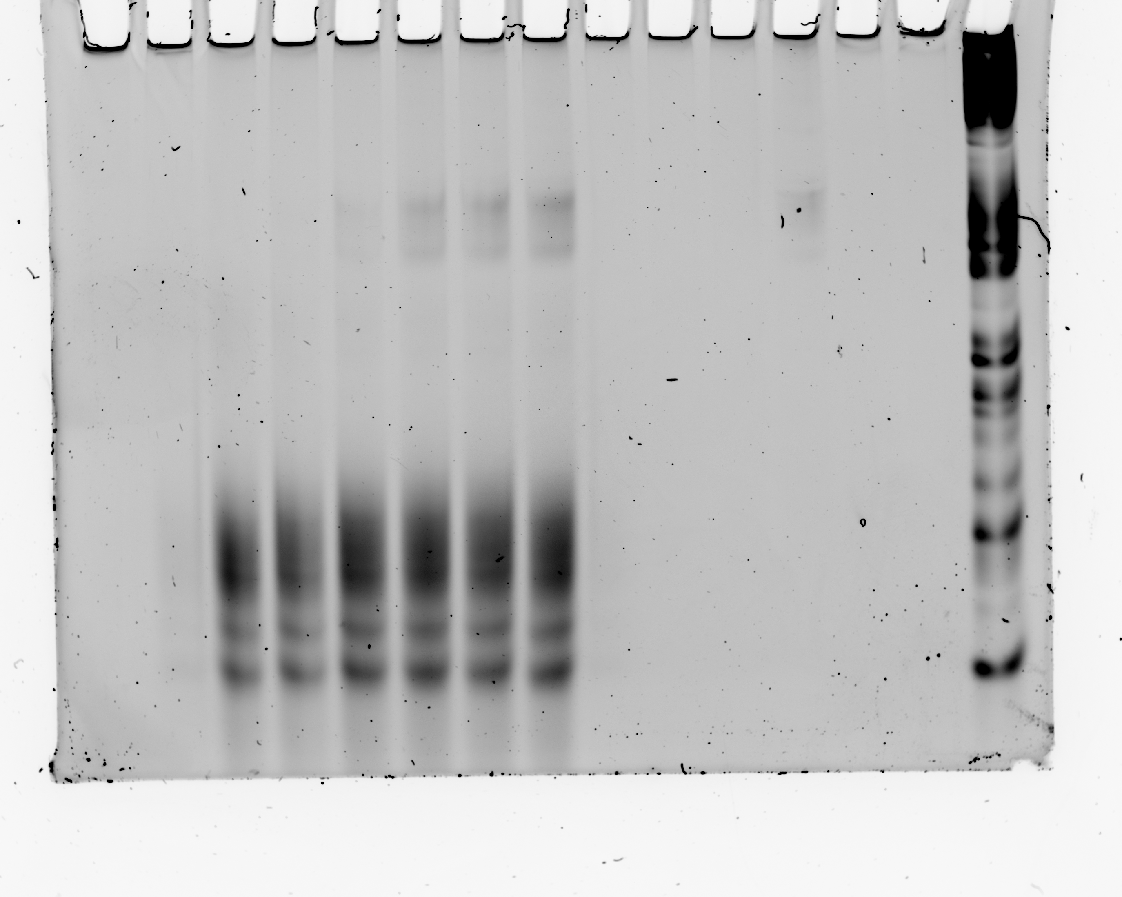

Supplement: Figure 5—source data 2. [file elife-101929-fig5-data2.zip › Figure5-SourceData-2/Schoggins Lab 2023-12-29 18h17m53s(SYBR® Gold).tif]

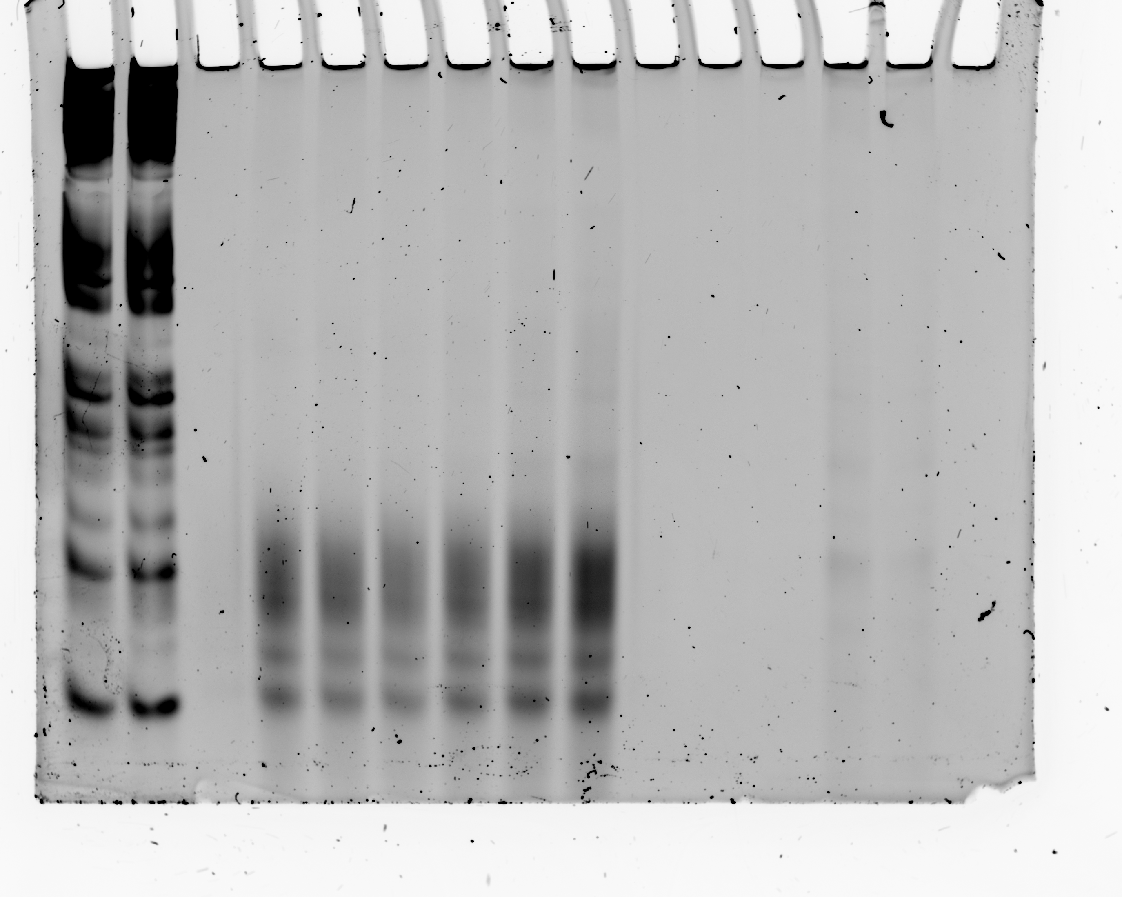

Supplement: Figure 5—source data 2. [file elife-101929-fig5-data2.zip › Figure5-SourceData-2/Schoggins Lab 2023-12-29 18h32m48s(SYBR® Gold).tif]

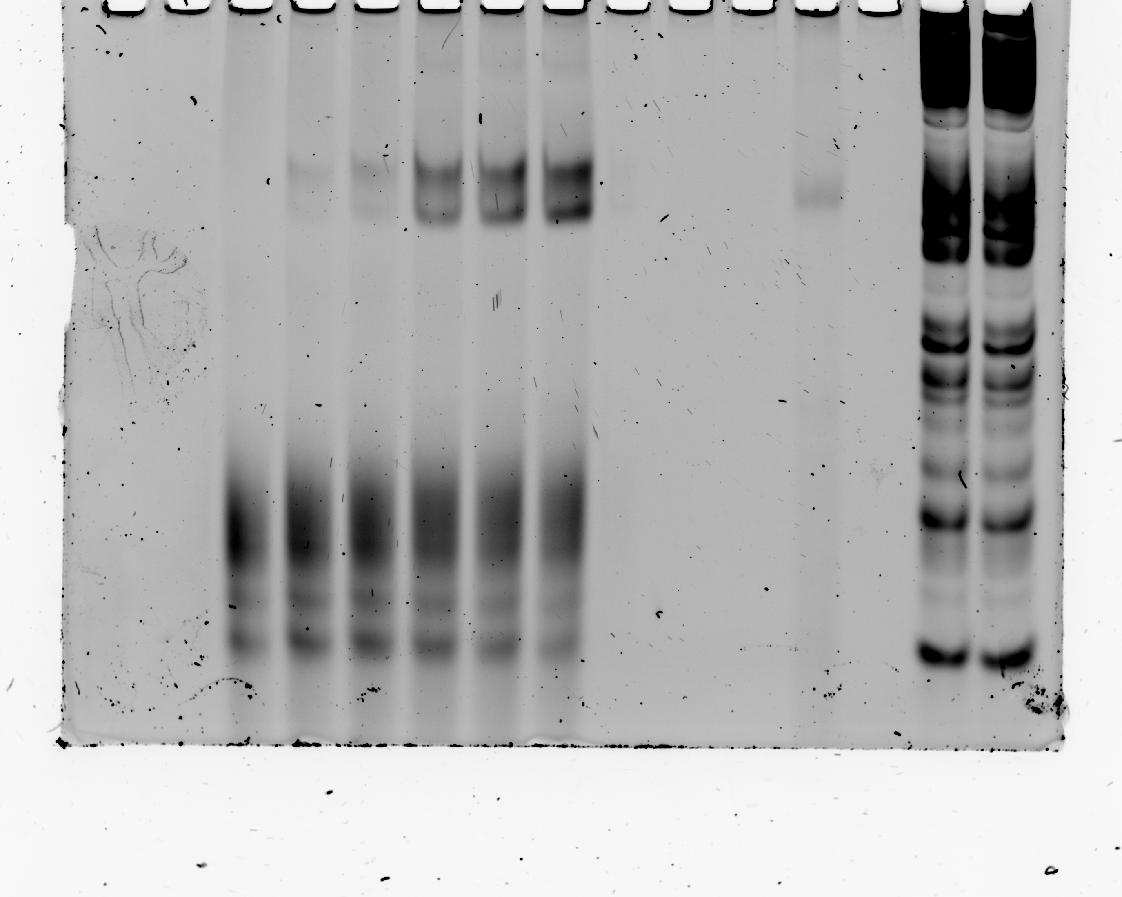

Supplement: Figure 5—source data 2. [file elife-101929-fig5-data2.zip › Figure5-SourceData-2/Schoggins Lab 2023-12-29 18h38m54s(SYBR® Gold).tif]

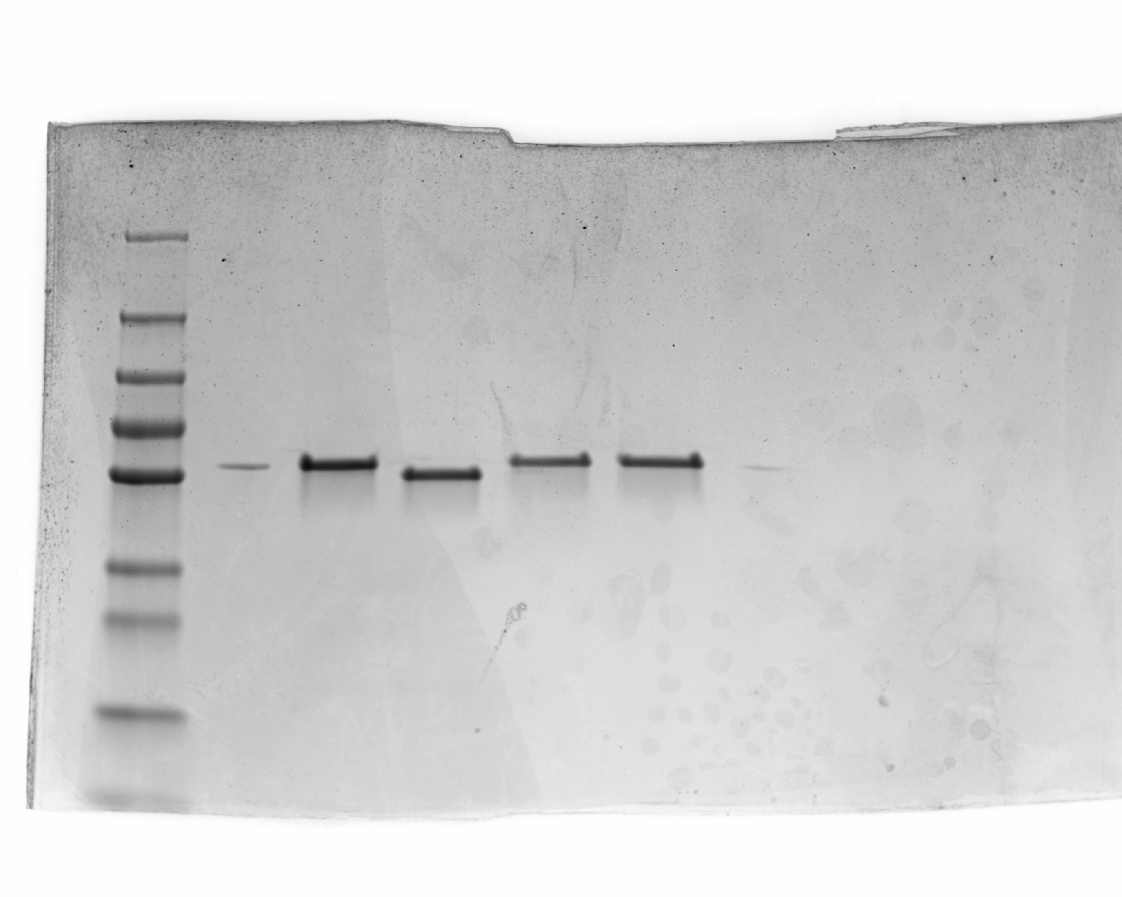

Supplement: Figure 5—figure supplement 1—source data 2. [file elife-101929-fig5-figsupp1-data2.zip › Figure5-FigureSupplement1-SourceData-2/Schoggins Lab 2024-04-25 12h46m59s(Coomassie Blue).tif]

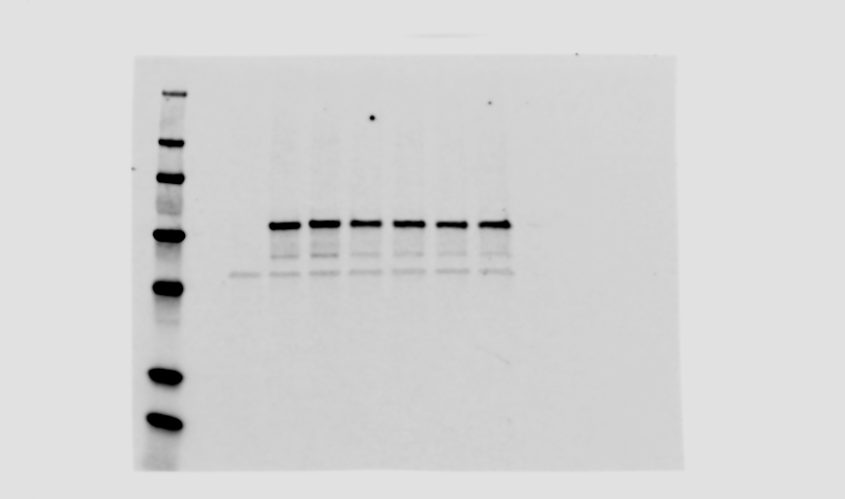

Supplement: Figure 6—source data 2. [file elife-101929-fig6-data2.zip › Figure6-SourceData-2/700 HA-tag - dblandtrplmut.tif]

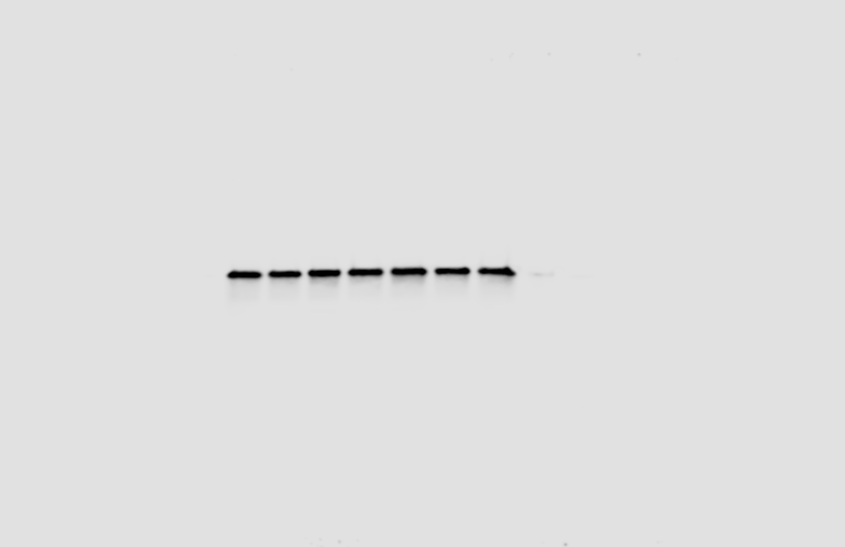

Supplement: Figure 6—source data 2. [file elife-101929-fig6-data2.zip › Figure6-SourceData-2/800 GAPDH - dblandtrplmut.tif]

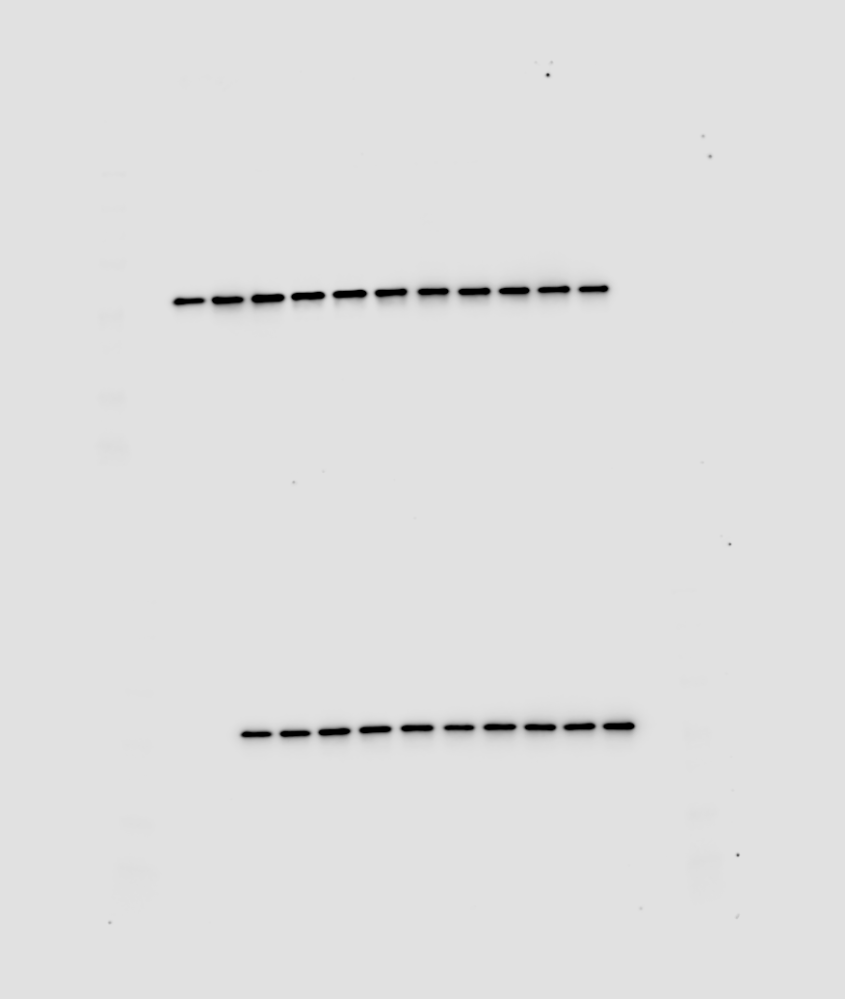

Supplement: Figure 6—source data 2. [file elife-101929-fig6-data2.zip › Figure6-SourceData-2/antiGAPDH.IFIT1.Top.EmptoHs414.Bottom.PtWTtpPt414.tif.tif]

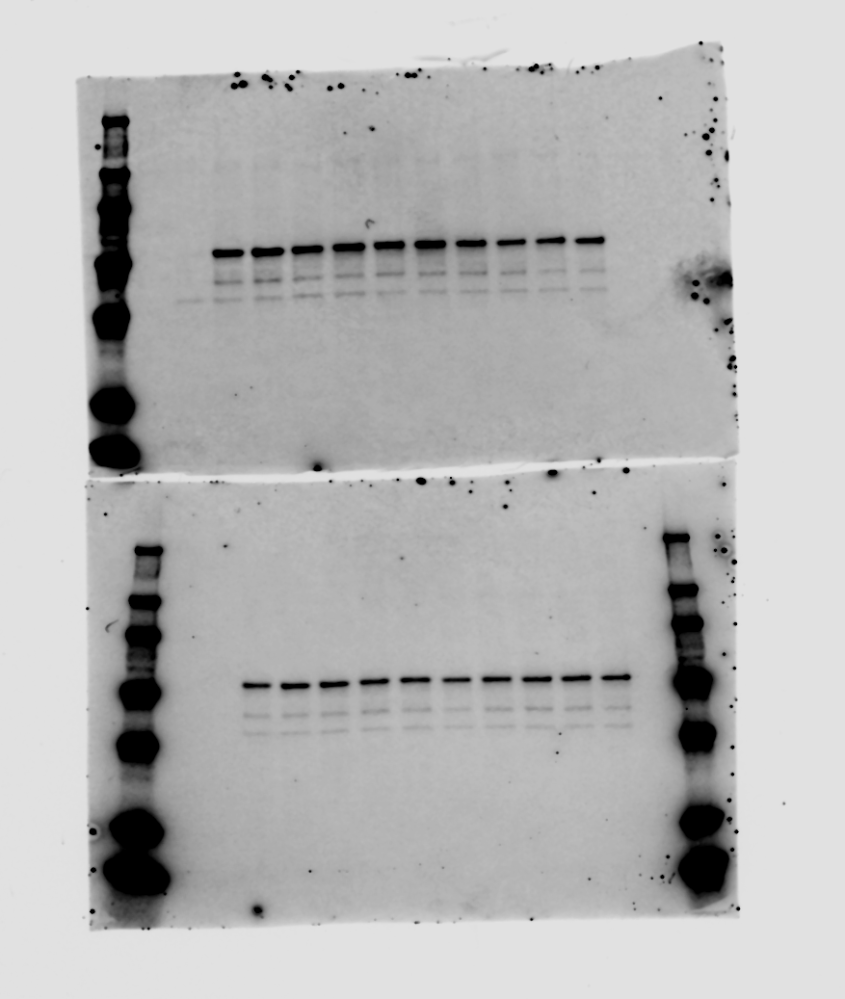

Supplement: Figure 6—source data 2. [file elife-101929-fig6-data2.zip › Figure6-SourceData-2/antiHA.IFIT1.Top.EmptoHs414.Bottom.PtWTtpPt414.tif]
