# Supplementary material for: IFIT1 is rapidly evolving and exhibits disparate antiviral activities across 11 mammalian orders
Source: eLife. 2025 Oct 22;13:RP101929. doi: 10.7554/eLife.101929 (PMC12543323; doi:10.7554/eLife.101929)
Supplement: Figure 5—source data 1. [file elife-101929-fig5-data1.zip › Figure5-SourceData-1.pdf]

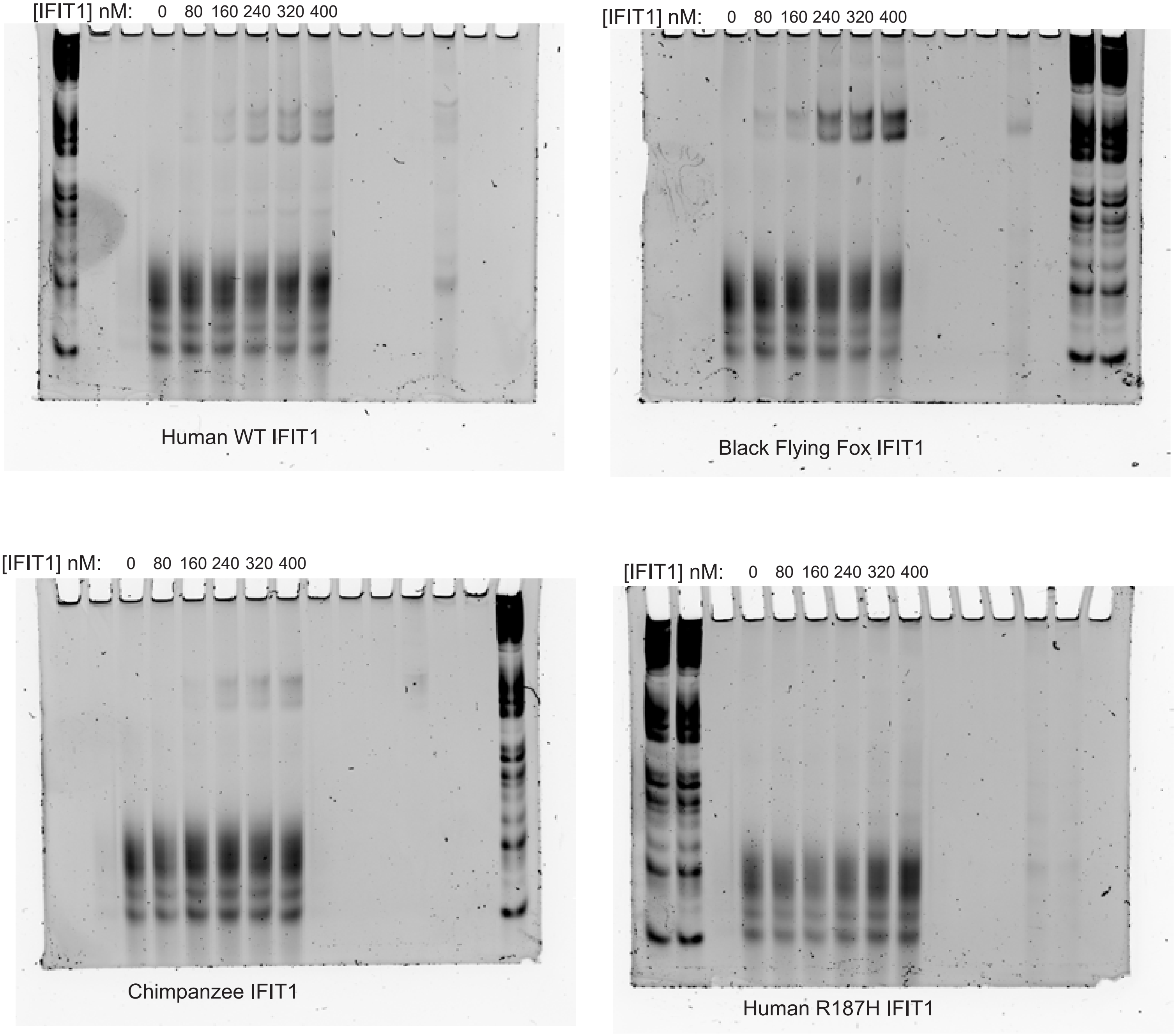

Figure 5- Source Data 1. Original images of SYBR Gold stained native gels corresponding to Figure 5, panel A.
