# Supplementary material for: IFIT1 is rapidly evolving and exhibits disparate antiviral activities across 11 mammalian orders
Source: eLife. 2025 Oct 22;13:RP101929. doi: 10.7554/eLife.101929 (PMC12543323; doi:10.7554/eLife.101929)
Supplement: Figure 5—figure supplement 1—source data 1. — Molecular weight was determined by PageRuler Plus Prestained Protein Ladder (Thermo Scientific). [file elife-101929-fig5-figsupp1-data1.zip › Figure5-FigureSupplement1-SourceData-1.pdf]

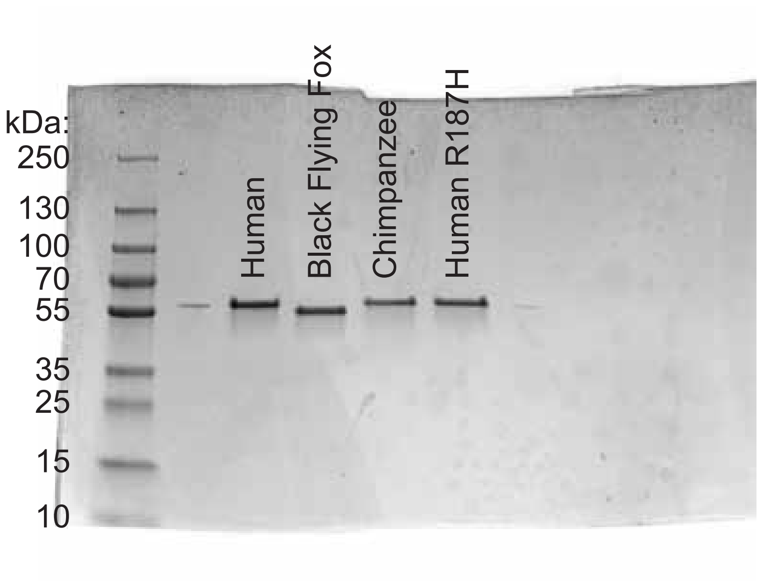

Figure 5-Figure Supplment 1-Source Data 1. Original image of Coomassie stained SDS-PAGE gel corresponding to Figure 5-Figure Supplement 1.
Molecular weight was determined by PageRuler Plus Prestained Protein Ladder (Thermo Scientific).
