# Supplementary material for: IFIT1 is rapidly evolving and exhibits disparate antiviral activities across 11 mammalian orders
Source: eLife. 2025 Oct 22;13:RP101929. doi: 10.7554/eLife.101929 (PMC12543323; doi:10.7554/eLife.101929)
Supplement: Figure 6—source data 1. — Molecular weight was determined by PageRuler Plus Prestained Protein Ladder (Thermo Scientific). [file elife-101929-fig6-data1.zip › Figure6-SourceData-1.pdf]

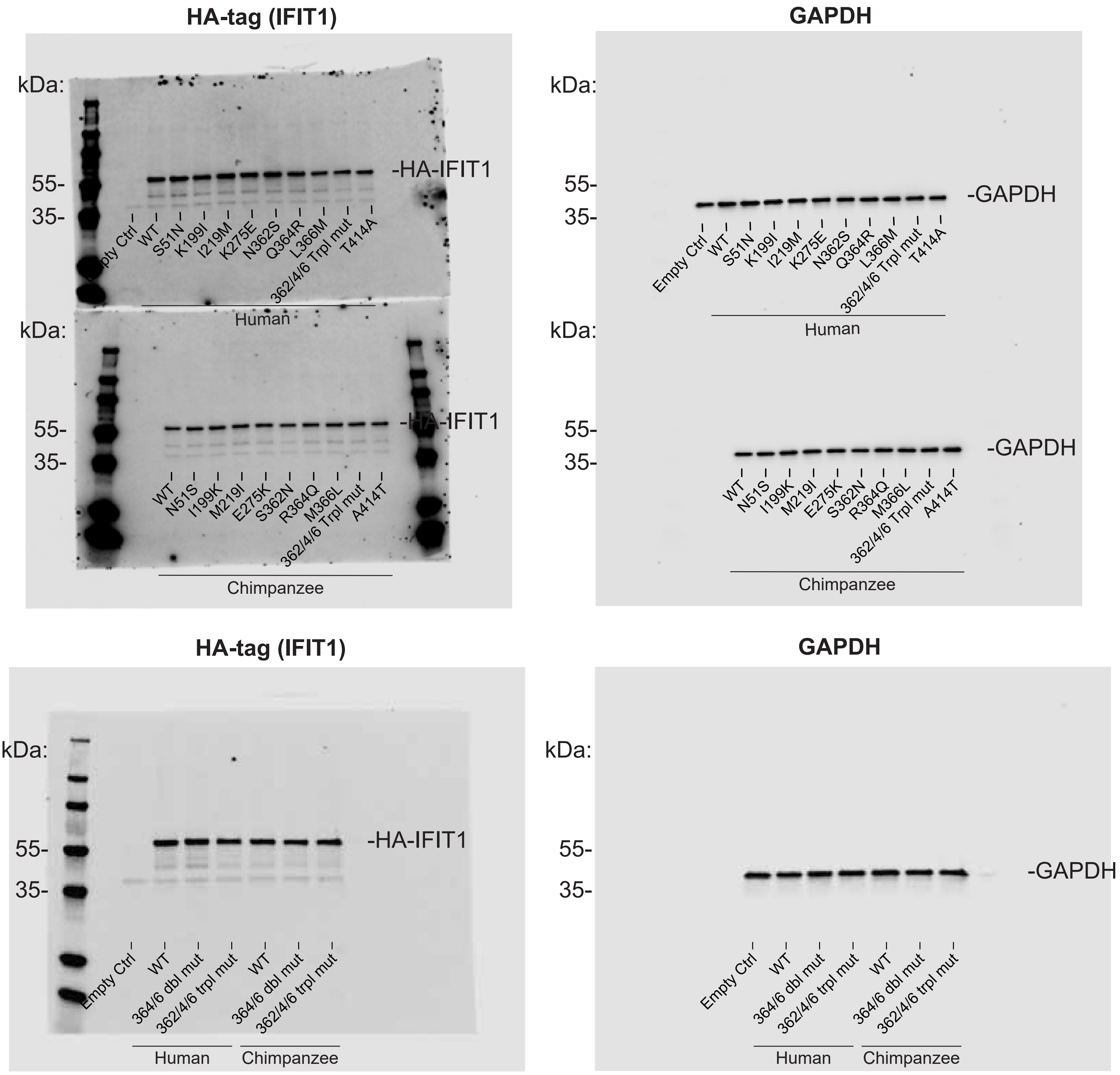

Figure 6- Source Data 1. Original images of membranes corresponding to Figure 6, panel B (top), and Figure 6, panel D (bottom).
Molecular weight was determined by PageRuler Plus Prestained Protein Ladder (Thermo Scientific).
